# Supplementary material for: Morphological, molecular, and pathological characterization of didymozoid trematode infection in the nasal cavity of orange-spotted grouper (Epinephelus coioides) from Arabian Gulf waters
Source: PLoS One. 2026 May 8;21(5):e0343608. doi: 10.1371/journal.pone.0343608 (PMC13155554; doi:10.1371/journal.pone.0343608)
Supplement: S1 File — (DOCX) [file pone.0343608.s001.docx]

**Summer (n = 14 infected fish)**

| **Fish ID** | **Cysts/nostril** | **Mean cyst diameter (mm)** |
| --- | --- | --- |
| S1 | 2 | 2.1 |
| S2 | 3 | 2.3 |
| S3 | 3 | 2.4 |
| S4 | 4 | 2.6 |
| S5 | 3 | 2.2 |
| S6 | 2 | 2.0 |
| S7 | 3 | 2.5 |
| S8 | 4 | 2.7 |
| S9 | 3 | 2.4 |
| S10 | 3 | 2.6 |
| S11 | 5 | 2.8 |
| S12 | 2 | 2.1 |
| S13 | 4 | 2.5 |
| S14 | 3 | 2.4 |

Mean cysts/nostril: **3.1 ± 0.8**
 Mean diameter : **2.4 ± 0.3 mm**

**Autumn (n = 16 infected fish)**

| **Fish ID** | **Cysts/nostril** | **Mean cyst diameter (mm)** |
| --- | --- | --- |
| A1 | 2 | 2.0 |
| A2 | 3 | 2.3 |
| A3 | 3 | 2.6 |
| A4 | 4 | 2.9 |
| A5 | 2 | 2.4 |
| A6 | 3 | 2.5 |
| A7 | 3 | 2.7 |
| A8 | 4 | 2.8 |
| A9 | 3 | 2.6 |
| A10 | 2 | 2.1 |
| A11 | 5 | 3.0 |
| A12 | 3 | 2.5 |
| A13 | 4 | 2.9 |
| A14 | 3 | 2.4 |
| A15 | 2 | 2.2 |
| A16 | 4 | 2.8 |

Mean cysts/nostril :**2.9 ± 0.7**
Mean diameter :**2.5 ± 0.4 mm**

**Winter (n = 13 infected fish)**

| **Fish ID** | **Cysts/nostril** | **Mean cyst diameter (mm)** |
| --- | --- | --- |
| W1 | 2 | 2.0 |
| W2 | 3 | 2.2 |
| W3 | 4 | 2.4 |
| W4 | 3 | 2.3 |
| W5 | 2 | 2.1 |
| W6 | 3 | 2.5 |
| W7 | 4 | 2.6 |
| W8 | 5 | 2.9 |
| W9 | 3 | 2.3 |
| W10 | 2 | 2.0 |
| W11 | 4 | 2.7 |
| W12 | 3 | 2.4 |
| W13 | 4 | 2.5 |

Mean cysts/nostril: **3.2 ± 0.9**
Mean diameter :**2.3 ± 0.3 mm**

**Spring (n = 9 infected fish)**

| **Fish ID** | **Cysts/nostril** | **Mean cyst diameter (mm)** |
| --- | --- | --- |
| SP1 | 2 | 2.0 |
| SP2 | 3 | 2.3 |
| SP3 | 2 | 2.4 |
| SP4 | 3 | 2.6 |
| SP5 | 4 | 3.0 |
| SP6 | 3 | 2.7 |
| SP7 | 2 | 2.2 |
| SP8 | 3 | 2.8 |
| SP9 | 3 | 2.5 |

Mean cysts/nostril: **2.7 ± 0.6**
Mean diameter: **2.6 ± 0.5 mm**
