# Supplementary figures and images for: Morphological, molecular, and pathological characterization of didymozoid trematode infection in the nasal cavity of orange-spotted grouper (Epinephelus coioides) from Arabian Gulf waters
Source: PLoS One. 2026 May 8;21(5):e0343608. doi: 10.1371/journal.pone.0343608 (PMC13155554; doi:10.1371/journal.pone.0343608)

## Slide 1
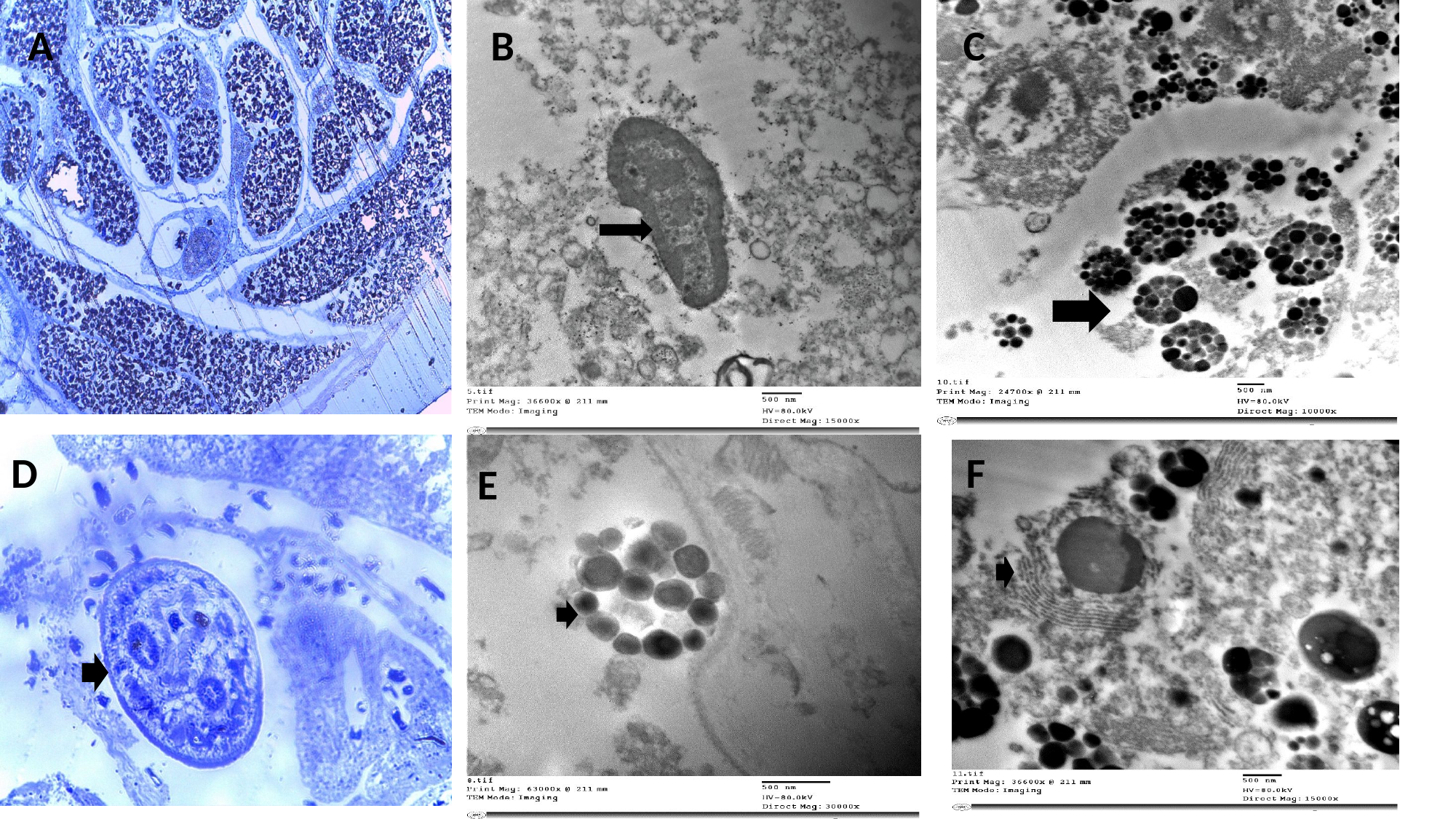

A
B
C
D
F
E

## Slide 2
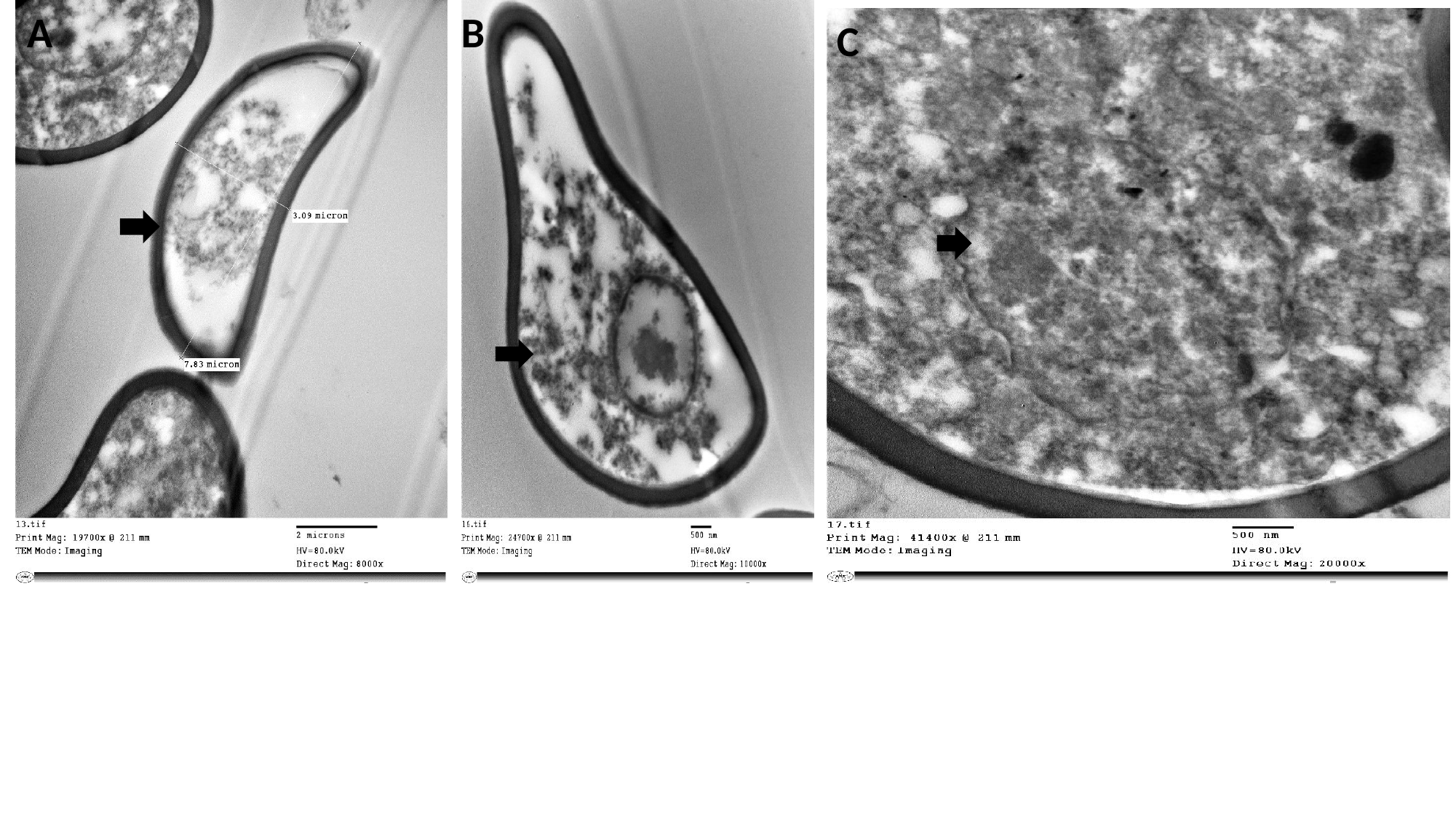

A
B
C

Supplement: S3 File — (PPTX) [file pone.0343608.s003.pptx]
