# Supplementary material for: Morphological, molecular, and pathological characterization of didymozoid trematode infection in the nasal cavity of orange-spotted grouper (Epinephelus coioides) from Arabian Gulf waters
Source: PLoS One. 2026 May 8;21(5):e0343608. doi: 10.1371/journal.pone.0343608 (PMC13155554; doi:10.1371/journal.pone.0343608)
Supplement: Attachment — Submitted filename: reviewer report.docx [file pone.0343608.s004.docx]

**Article:** PONE-D-25-51753

**Title:** Pathological, morphological, and molecular analysis of didymozoid trematodes infection in the nasal cavity of orange-spotted grouper (*Epinephelus coicoides*) caught from Arabian Gulf waters.

**Reviewer report:**

In general, this is a well-organized and very interesting manuscript. The authors have analyzed three categories of parasitology, i.e. pathological, morphological and molecular level investigations, for Didymozoidae in orange-spotted grouper hosts, and generated a comprehensive report. However, there are few questions need to be answered. If the authors can adequately answer these questions, I will recommend accept the manuscript for publication.

1. Regarding the histopathological analyses (lines 133-137), when you classified tissues as infected, did you examine the entire nasal cavity, or just specific portions of the nasal chamber? How did you determine which infected tissues for sampling, particularly in terms of their size and shape? What was your protocol for processing these tissue samples for the subsequent histological examinations?

2. For the molecular identification of the parasites, I could not find any information about your biological and technical replicates. To obtain a statistically valid picture of genetic variation, you need to balance breadth (sampling different hosts from different locations) with depth (analyzing multiple parasites from each host). So, how many individual worms did you collect from each host? And, how many hosts in total were sampled for rDNA sequencing? Did you pool samples from multiple worms together, or was each worm sequenced individually? Also, how many sequencing replicates did you perform for each sample to ensure reproducibility?

3. The phylogenetic analysis relies exclusively on the 28S rDNA D1 - D3 regions. However, using solely this molecular marker may be problematic:

1. Many didymozoids have undergone recent divergence as they adapted to specific microhabitats within particular fish hosts, which result in cryptic species complexes (rDNA sequences suggest organisms are the same, while their biological characteristics indicate they are different).

2. If these parasites evolved rapidly, it creates rapid radiation. In such cases, the branches representing different species in a 28S rDNA phylogenetic tree become extremely short, making it difficult to resolve relationships between closely related species.

3. Some didymozoids possess unusually long expansion segments in their 28S rDNA, (especially D2 and D3). In such lineages, these expansion segments can evolve at very slow substitution rates, resulting in highly conserved LSU sequences across multiple species. This conservation means different species may share identical or nearly identical D1 - D3 sequences, limiting resolution to the genus level rather than enabling species level discrimination. Moreover, when LSU sequences are extremely conserved, D1 - D3 sequencing lacks sufficient variation to resolve fine scale evolutionary divergences, such as population level genetic structure.

Given these limitations, what is your rationale for concluding that 28S D1 - D3 rDNA sequencing alone provides sufficient resolution to construct a comprehensive phylogenetic framework for Didymozoidae? Will you consider a multi marker approach, such as ITS2 (rDNA spacer, which help resolving species/sibling species relationships) and COI (mtDNA, which help detecting shallow divergences and identify cryptic populations), to create a concatenated phylogenetic tree would provide a more complete picture of Didymozoidae evolutionary history?
