# Supplementary material for: Morphological, molecular, and pathological characterization of didymozoid trematode infection in the nasal cavity of orange-spotted grouper (Epinephelus coioides) from Arabian Gulf waters
Source: PLoS One. 2026 May 8;21(5):e0343608. doi: 10.1371/journal.pone.0343608 (PMC13155554; doi:10.1371/journal.pone.0343608)
Supplement: Attachment — Submitted filename: Response to Reviewers.docx [file pone.0343608.s006.docx]

**Response to Reviewers' Comments**

**Article:** PONE-D-25-51753

**Original Title:** Pathological, morphological, and molecular analysis of didymozoid trematodes infection in the nasal cavity of orange-spotted grouper (*Epinephelus coioides*) caught from Arabian Gulf waters.

**Revised title:** "Morphological, molecular, and pathological characterization of didymozoid trematode infection in the nasal cavity of orange-spotted grouper (*Epinephelus coioides*) from Arabian Gulf waters"

We sincerely thank both reviewers for their constructive feedback and insightful comments, which have significantly improved the quality of our manuscript. Below, we provide detailed point-by-point responses to all comments raised.

# Reviewer 1:

We thank Reviewer 1 for the thorough evaluation of our manuscript and for providing valuable suggestions that have helped us improve the clarity and completeness of our work. We have addressed all comments as detailed below.

## Comment 1:

*Line 128 and 133: Why were the didymozoid cysts fixed in 5% neutral buffered formol saline instead of 10% neutral buffered formalin, as used for the infected tissues?*

**Response:**

We appreciate the reviewer's attention to this methodological detail. The didymozoid cysts were fixed in 5% neutral buffered formol saline to preserve the delicate cyst capsule structure without over-hardening for 24 hours, for parasitological identification. In contrast, the infected tissues for histopathological examination were fixed in standard 10% neutral buffered formalin to ensure optimal tissue preservation and fixation for routine histological processing and staining. We have clarified this methodological distinction in the revised Materials and Methods section.

## Comment 2:

*Line 165: Please specify the expected and/or obtained PCR product size.*

**Response:**

Thank you for this suggestion. The expected PCR product size for the 28S rDNA D1-D3 region amplification was approximately 1,000-1,100 bp. Our obtained PCR products were 1,042 bp, which is consistent with this expected range and confirms successful amplification of the target region. This information has been added to the Materials and Methods section.

## Comment 3:

*Line 196-197: Since no external signs of inflammation or tissue damage were observed around the cyst attachment sites, does the relationship between the didymozoid trematodes and the orange-spotted groupers represent a parasitic, mutualistic, or commensal association?*

**Response:**

This is an excellent question that raises important considerations about host-parasite relationships. Actually, didymozoid trematodes are obligatory parasites infecting fish; sometimes, it encapsulated externally on nasal cavity (as recorded in our study) or inside the lamellar tissue of the gills, or even in fish musculature, as recoded by many other studies. In all cases, the constituents of the capsule (mainly connective tissue elements) are derived from the host tissue, with variable harmful effects on the sites of their attachment, depending on the severity of tissue reaction and pathogenicity of the parasite. This host-parasite interaction (parasitism) is differing from mutualistic (a relationship where both organisms benefit from living together), and commensal relationships (a relationship where one organism benefits, while the other is neither significantly helped nor harmed). Fish parasites, particularly certain helminth larvae, can be encapsulated by fibrous capsules without inducing a strong, immediate inflammatory reaction. This mechanism often allows the parasite to remain viable for long periods. While some encapsulation acts as a defence to limit tissue damage, it often represents a passive co-existence.

## Comment 4:

*Line 2: Since no external signs of inflammation or tissue damage were observed, the authors may need to reconsider the use of the terms "pathological" and "infection" in the title.*

**Response:**

We appreciate the reviewer's concern about terminology accuracy. We have changed the title as following:"Morphological, molecular, and histopathological characterization of didymozoid trematode infection in the nasal cavity of orange-spotted grouper (Epinephelus coioides) from Arabian Gulf waters".

We have replaced the broader term "pathological" with the more precise term "histopathological," which accurately reflects our systematic microscopic examination of tissue-level changes. Regarding the term "infection," we respectfully maintain its use as it is appropriate from a parasitological perspective. Didymozoids are obligate parasites that invade and establish residence in host tissues, constituting a true parasitic infection by standard parasitological definition, regardless of pathology severity.

## Comment 5:

*Is there any data available on the physiological parameters (weights and lengths) of orange-spotted groupers with and without didymozoid cysts? Such information could help determine whether the presence of didymozoid cysts affects the growth and health of orange-spotted groupers.*

**Response:**

This is an excellent suggestion that would have provided valuable insights into the impact of didymozoid infection on host fitness. Unfortunately, we did not systematically collect morphometric data (weight and length measurements) comparing infected versus uninfected fish during this study. We fully agree that such comparative analyses of condition factors, growth parameters, and overall health indices would be valuable for assessing the biological significance of these parasitic infections. We have incorporated this recommendation into our future research directions in the revised manuscript.

## Comment 6:

*Line 58-66: Please provide more information about the family Didymozoidae and didymozoid trematodes, including aspects such as their pathogenesis, impact on the host, and associated mortality in host.*

**Response:**

We thank the reviewer for this important suggestion. We have substantially expanded the Introduction section to provide comprehensive background information on Didymozoidae biology and pathological impacts (lines 96-110). These additions provide readers with essential context for understanding the biological and economic importance of didymozoid in Arabian Gulf waters.

# Reviewer 2

We are grateful to Reviewer 2 for the detailed and constructive evaluation of our manuscript. The reviewer's expertise in molecular parasitology and integrative taxonomy has been invaluable in strengthening our work. We have carefully addressed all comments and believe the manuscript is substantially improved as a result.

## Comment 1:

*Regarding the histopathological analyses (lines 133-137), when you classified tissues as infected, did you examine the entire nasal cavity, or just specific portions of the nasal chamber? How did you determine which infected tissues for sampling, particularly in terms of their size and shape? What was your protocol for processing these tissue samples for the subsequent histological examinations?*

**Response:**

We appreciate the reviewer's request for clarification on our histopathological sampling methodology; the existed cysts containing the trematodes were detached carefully and fixed in the proper fixative for further tissue processing. We agree with you in that, the site of attachment on the nasal surface were very difficult to recognize grossly for tissue sampling. So, the detection of these sites of parasites’ attachment was easily recognized on the surface of the cysts, where remnants of the nasal tissue were detached and adhered on the cyst. In this regard, the characteristic epithelial cells of the nasal tissue were clearly demonstrated in Fig. 2B (See letters, ht). So, further future studies are required on the life cycle and pathogenesis of this parasite to determine the specific sites of their attachment on nasal mucosa, factors influencing this attachment process and microscopic tissue alterations in whole nasal tissue of the host.

## Comment 2:

*For the molecular identification of the parasites, I could not find any information about your biological and technical replicates. To obtain a statistically valid picture of genetic variation, you need to balance breadth (sampling different hosts from different locations) with depth (analyzing multiple parasites from each host). So, how many individual worms did you collect from each host? And, how many hosts in total were sampled for rDNA sequencing? Did you pool samples from multiple worms together, or was each worm sequenced individually? Also, how many sequencing replicates did you perform for each sample to ensure reproducibility?*

**Response:**

We thank the reviewer for highlighting the importance of clearly documenting our sampling strategy and replication approach. We analyzed four individual didymozoid specimens from four different E. coioides hosts collected across different seasons—each processed separately, not pooled. PCR was performed in duplicate for each specimen, and all amplicons were bidirectionally sequenced. Sequence consistency between replicates was confirmed before analysis. All four specimens yielded high-quality sequences (1,042 bp, GenBank: PQ736510-PQ736513), revealing two haplotypes (99.81% identity). Our seasonal sampling strategy prioritized breadth to establish baseline molecular identification for this previously uncharacterized system and detect temporal variation. We acknowledge that deeper within-host sampling would strengthen population genetics analyses and have added this to our limitations section.

## Comment 3:

*The phylogenetic analysis relies exclusively on the 28S rDNA D1-D3 regions. However, using solely this molecular marker may be problematic: (1) Many didymozoids have undergone recent divergence as they adapted to specific microhabitats within particular fish hosts, which result in cryptic species complexes (rDNA sequences suggest organisms are the same, while their biological characteristics indicate they are different). (2) If these parasites evolved rapidly, it creates rapid radiation. In such cases, the branches representing different species in a 28S rDNA phylogenetic tree become extremely short, making it difficult to resolve relationships between closely related species. (3) Some didymozoids possess unusually long expansion segments in their 28S rDNA (especially D2 and D3). In such lineages, these expansion segments can evolve at very slow substitution rates, resulting in highly conserved LSU sequences across multiple species. This conservation means different species may share identical or nearly identical D1-D3 sequences, limiting resolution to the genus level rather than enabling species-level discrimination. Moreover, when LSU sequences are extremely conserved, D1-D3 sequencing lacks sufficient variation to resolve fine-scale evolutionary divergences, such as population-level genetic structure. Given these limitations, what is your rationale for concluding that 28S D1-D3 rDNA sequencing alone provides sufficient resolution to construct a comprehensive phylogenetic framework for Didymozoidae? Will you consider a multi-marker approach, such as ITS2 (rDNA spacer, which help resolving species/sibling species relationships) and COI (mtDNA, which help detecting shallow divergences and identify cryptic populations), to create a concatenated phylogenetic tree would provide a more complete picture of Didymozoidae evolutionary history?*

**Response:**

We acknowledge these important limitations of single-marker phylogenetics. The 28S rDNA marker was selected based on established use in didymozoid systematics and available reference sequences for comparative analysis. While this marker successfully placed our specimens within Didymodiclinus (99.52% similarity to congeneric species) and revealed intraspecific variation (two haplotypes, 99.81% identity), we agree it has known limitations for resolving closely related species and detecting cryptic diversity. We have revised our discussion to explicitly address these constraints and clarify that our analysis provides robust genus-level identification and phylogenetic placement rather than definitive species delimitation. We now recommend multi-marker approaches (ITS2, COI) as essential future work for fine-scale population structure assessment and cryptic species detection. This recommendation has been added to both the discussion and study limitations sections, emphasizing that integrating multiple loci would provide the resolution necessary to address the specific challenges you outline regarding rapid radiation, conserved expansion segments, and cryptic species complexes in Didymozoidae.
